# Educational Review: Management of Lymphedema—Approaches, Evidence for Surgical and Nonsurgical Interventions

**DOI:** 10.1245/s10434-025-17463-x

**Published:** 2025-05-20

**Authors:** Lavinia M. Anzai, David W. Chang

**Affiliations:** Department of Surgery, Section of Plastic and Reconstructive Surgery, University of Chicago Medicine, Chicago, IL USA

## Abstract

**Background:**

We aim to review the current medical and surgical management strategies for lymphedema and present methods for their successful integration into clinical practice. In addition, the following summary background data is provided. Lymphedema is a chronic condition resulting from impaired lymphatic drainage, leading to significant morbidity and reduced quality of life. Traditional management includes conservative therapies such as compression garments and physical therapy. However, advancements in surgical techniques have opened new avenues for treatment. This review aims to synthesize the available literature on both medical and surgical approaches to lymphedema management.

**Materials and Methods:**

This article explores the senior author’s strategies and experiences in lymphedema treatment, supplemented by a literature review that supports the described techniques.

**Results:**

Effective lymphedema management is multifaceted, necessitating accurate diagnosis, medical treatment, and in some cases, surgical intervention. The senior author has sought to streamline his approach to this complex condition, and this article outlines his algorithm and techniques for treatment.

**Conclusions:**

While conservative treatments remain the foundation of lymphedema management, surgical options in the properly selected patients have shown promising results. The integration of these approaches could enhance overall treatment efficacy and improve quality of life for individuals affected by lymphedema.

Lymphedema is a chronic and debilitating condition characterized by the accumulation of lymphatic fluid, most commonly in the extremities, leading to swelling, limb heaviness, and susceptibility to infections, among other uncomfortable pathologies. This condition can develop in two main forms: primary lymphedema, which is typically caused by genetic factors, and secondary lymphedema, which often results from trauma, surgical procedures, or radiation therapy, especially related to cancer treatment.

The treatment of lymphedema can be separated into medical and surgical arms, which in the ideal setting, work synergistically to improve quality of life of patients with this debilitating condition. Medical therapies consist of a combination of weight management, physical therapy, compression garments, and avoidance of infection, while surgical options can include physiological and debulking procedures. With advancements in our understanding of lymphedema, we have enhanced our ability to provide a wider range of treatments and improve patient outcomes. This review aims to synthesize current knowledge regarding the diagnosis and treatment options for lymphedema, highlighting recent therapeutic advances and clinical practices.

## Lymphatic Imaging

The lymphatic system plays a crucial role in maintaining fluid balance, immune function, and the transport of nutrients throughout the body. Novel imaging technologies have revolutionized our ability to visualize and understand this complex network. Radionuclide lymphoscintigraphy, first introduced in 1953, has long been considered the gold standard in lymphedema diagnosis. This study involves the use of radioactive tracers to visualize the lymphatic system and assess its function. During the procedure, a small amount of a radioactive substance, Technetium-99, is injected into the tissue near the area of interest. The tracer is then taken up by the lymphatic vessels, allowing for the visualization of lymphatic drainage patterns through a gamma camera. The transport index (TI) is a semiquantitative analysis that can be used to determine the degree of lymphedema and that serves as a metric to follow its progression or improvement over time.^1^ Despite its advantages, lymphoscintigraphy has several drawbacks: (1) images are captured at standardized timepoints, often spanning several hours, which can be time-consuming; (2) the procedure does not give good information on the anatomy of the lymphatic channels, and therefore provides minimal guidance for surgical planning with respect to lymphovenous bypasses; (3) image quality can be poor and of low resolution; and (4) the injection may cause discomfort for patients.^2^ In light of these shortcomings, new imaging modalities have emerged as alternatives for diagnosing lymphedema.

**Fig. 1:**
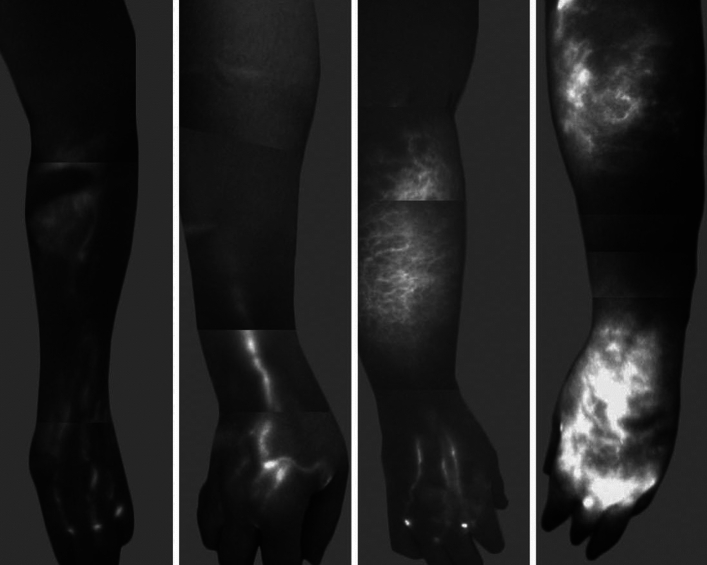
M.D. Anderson lymphedema classification based on indocyanine green lymphangiographic findings: stage 1 (left): many patent lymphatic vessels, with minimal, patchy dermal backflow; stage 2 (second from the left): moderate number of patent lymphatic vessels, with segmental dermal backflow; stage 3 (second from the right): few patent lymphatic vessels, with extensive dermal backflow involving the entire arm; and stage 4 (right): no patent lymphatic vessels seen, with severe dermal backflow involving the entire arm and extending to the dorsum of the hand; *source*: Chang DW, Suami H, Skoracki R. A prospective analysis of 100 consecutive lymphovenous bypass cases for treatment of extremity lymphedema. *Plast Reconstr Surg*. 2013;132(5):1305–1314. https://doi.org/10.1097/PRS.0b013e3182a4d626

The emergence of physiologic surgeries for lymphedema has sparked a growing interest in the development of imaging modalities that can accurately pinpoint functional lymphatic channels. Indocyanine green lymphography (ICG-L) has gained traction as a valuable tool for identifying the location of functional lymphatic vessels, which, in turn, aids in determining the optimal sites for performing lymphovenous bypasses (LVB). This procedure provides a real-time assessment of the lymphatic system using a near-infrared camera, and the severity of lymphedema can be graded according to the diffusion pattern of ICG seen^2^ (Fig. 1). ICG-L has revolutionized the surgeon’s approach to lymphovenous bypass, increasing operative efficiency and drastically simplifying the procedure. Still, ICG-L is limited in that it is unable to visualize the deep lymphatic circulation and does not provide information on changes in the limb.

More recently, there has been a growing interest in the use of ultra-high-frequency ultrasound (UHFUS) as an effective tool for improving lymphatic vessel selection. ICG-L’s effectiveness can be limited in severe lymphedema cases, where dermal backflow patterns obscure lymphatic flow. UHFUS offers the advantage of visualizing functional lymphatic flow and valves, helping surgeons to assess the degenerative status of vessels and select the most suitable ones for anastomosis. As a result, UHFUS aids in identifying optimal sites for surgery, ultimately improving the accuracy and success of LVB. However, the technique’s wide adoption has been limited by its steep learning curve and high operator dependency.^3^

Magnetic resonance lymphangiography (MRL) is an advanced imaging technique that utilizes magnetic resonance imaging (MRI) to visualize the lymphatic system. One of its key advantages is the ability to produce detailed, high-resolution images of lymphatic structures without exposing patients to ionizing radiation. MRL also excels in providing soft tissue contrast, offering insights not only into the presence of functional lymphatics, but also into the characteristics of the affected limb and the status of any nodal basins. This information is invaluable for guiding surgical strategies, as it can help identify patients with significant limb fibrosis, who may be unsuitable candidates for physiological procedures due to their reduced likelihood of benefiting from such interventions.^4^ Despite its benefits, MRL is a time-consuming procedure that may be limited in availability in certain healthcare settings.

### Treatment

Optimal treatment for lymphedema begins with an accurate diagnosis as delineated above. Management of lymphedema includes both surgical and nonsurgical approaches, with the latter categorized into physiological and debulking procedures. Patients are initially referred for conservative management, and those who meet criteria may then proceed to surgical intervention.

### Nonsurgical Management

#### Pharmacologic Treatment

Currently, there is no evidence to support the use of medications for treating lymphedema. Various pharmaceutical agents, including diuretics, coumarin, vitamin E, and steroids, have been discussed, but none have demonstrated compelling efficacy in managing lymphedema.

While diuretics may reduce limb volume, this effect is largely anecdotal and likely results from their action on swelling caused by conditions such as heart failure or venous stasis, rather than from treating lymphedema itself.^5^ In fact, some authors have raised concern that diuretics may exacerbate the condition by increasing the concentration of high protein fluid in the interstitial space.^6,7^ Similarly, coumarin, vitamin E, and pentoxifylline have been explored for their potential in preventing or treating lymphedema, but there is no supporting evidence for their effectiveness.^8,9^ Steroids have been shown to have a temporary benefit in lymphedema treatment, however, this effect is short-lived, and was not shown to extend beyond a month.^10^

#### Complete Decongestive Therapy

Complete decongestive therapy (CDT) has been a mainstay in nonsurgical management since its introduction in the 1980s.^11^ Once a diagnosis of lymphedema is confirmed, patients are referred to a licensed lymphedema therapist who supervises the implementation of CDT, which includes manual lymphatic drainage (MLD), compression, exercise, skin care, and education. The success of this treatment largely depends on patient adherence to the regimen, which involves regular visits with the specialist divided into two phases. Phase I typically consists of an intensive 6-week program aimed at reducing the size of the swollen limb and preventing infection. Phase II follows, during which the focus shifts to maintenance therapy, promoting self-management for patients. They are encouraged to wear custom-fitted garments designed specifically for their limb to provide continuous compression and to maintain limb size.

CDT is often regarded as the first-line treatment for lymphedema, with studies revealing reduction in limb volumes and improvement in quality of life.^12–14^ There is ongoing debate in the literature regarding which components of complete decongestive therapy are essential for effective volume reduction. Some studies suggest that individual elements, such as compression, physical exercise, or manual lymphatic drainage, may be sufficient on their own.^15–17^ These studies, however, may be limited by small sample sizes. More recently, intermittent pneumatic compression (IPC) has been proposed as a viable adjunct to CDT for select patients with lymphedema. A 2024 systematic review assessing IPC found that, although its addition did not affect limb volume significantly, it significantly improved external joint mobility.^18^

#### Weight Loss and Exercise

Obesity has been shown to be a major risk factor in the development of lymphedema, with multiple studies showing that patients with a higher body mass index are more significantly more likely to develop lymphedema.^19–22^ It reasons that weight loss should be a beneficial adjunct in helping to reduce limb volume; however, this has not been borne out in the literature with certainty. A 2007 randomized controlled trial examined the impact of weight reduction on breast-cancer related lymphedema, with results showing significant reduction in arm volume in the weight-reduction group after 12 weeks; however, this study was limited by a small sample size.^23^ Since then, larger, more recent studies have reported fewer promising results, indicating that weight loss does not improve lymphedema symptoms. Schmitz et al. found in a large-scale randomized clinical trial that weight loss and exercise did not significantly improve breast-cancer-related lymphedema outcomes compared to the control group. ^24^ Similarly, a systematic review and meta-analysis found that weight loss did not reduce severity of breast-cancer-related lymphedema.^25^ It may be the patient’s weight at the time of surgery or lymphatic injury that determines their risk of lymphedema development, rather than their weight after the condition has already developed.

While the effectiveness of weight loss as a treatment for lymphedema is still debated, the role of exercise as a therapeutic approach is more supported. In general, exercise does not seem to worsen lymphedema symptoms, with literature suggesting that resistance training may be specifically beneficial.^26–28^ Aquatic activities have also been recommended. Movement in a water-based environment eliminates a portion of gravitational load while supplying external compression, which together aids in the movement of fluid proximally and out of the extremity.^29^

### Surgical Management

Once a patient’s medical therapy is optimized, they may be considered for surgical evaluation. Surgical options can be categorized into physiological and ablative procedures. Physiological procedures directly target the lymphatic system and include lymphovenous bypass and vascularized lymph node transfer. Ablative procedures focus on reducing limb size through debulking by means of liposuction or direct excision. Recent years have also seen a rise in interest in prophylactic procedures for lymphedema prevention.

### Physiological Procedures

#### Lymphovenous Bypass

Lymphovenous bypass (LVB) is designed to restore the flow of lymphatic fluid by creating a connection between lymphatic vessels and the venous system. By rerouting lymphatic fluid away from areas of congestion, LVB can reduce limb volume and improve overall limb function. As described above, ICG-L or UHFUS can be performed to easily determine a patient’s suitability for LVB by visualizing and identifying the location of lymphatic channels available for bypass. These imaging studies can be performed in the office or in the operating room, ensuring that surgeons can make informed decisions regarding the potential for successful surgical intervention.

**Fig. 2:**
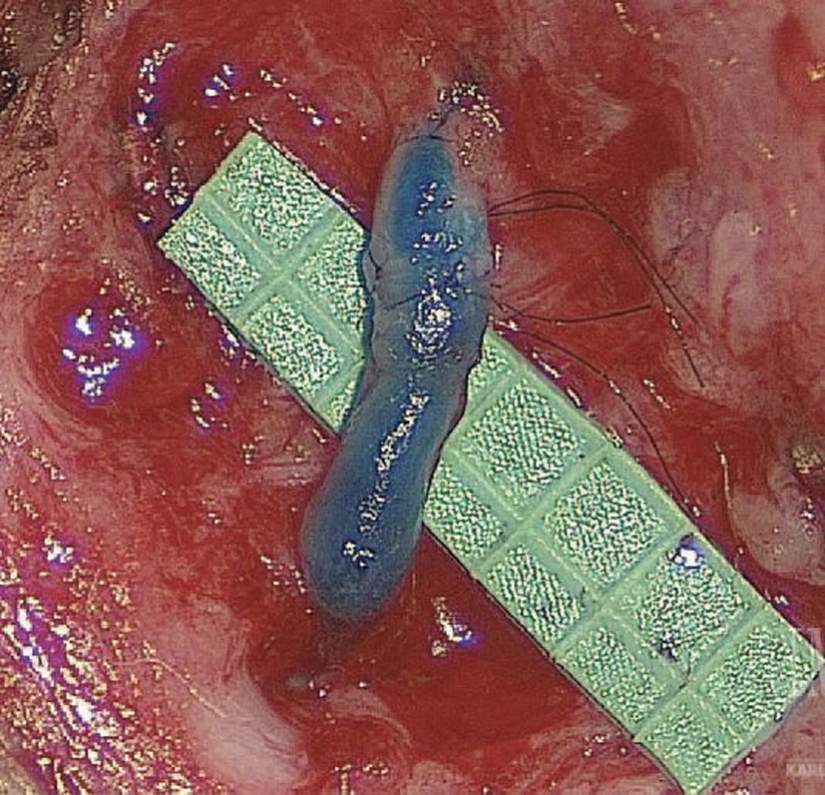
Patency of the bypasses is confirmed by observing the isosulfan blue dye or ICGN pass from the lymphatic vessel through the anastomosis and into the vein; *source*: Chang DW, Suami H, Skoracki R. A prospective analysis of 100 consecutive lymphovenous bypass cases for treatment of extremity lymphedema. *Plast Reconstr Surg*. 2013;132(5):1305–1314. https://doi.org/10.1097/PRS.0b013e3182a4d626

A transverse incision measuring approximately 2–3 cm is marked, and lidocaine with epinephrine is injected into the area, taking care to keep the infiltration superficial within the dermis. The entire procedure is performed under a high-powered microscope with fluorescent capabilities to aid in the identification of the targeted lymphatic channel. The channel is recognized by its blue dye and fluorescence under infrared imaging. Once located, the lymphatic channel is isolated, dissected, and transected distally. The surgeon then searches for a vein of similar caliber within the wound bed and carefully dissects it. Ideally, the vein should match in size and exhibit minimal to no backflow to facilitate a successful end-to-end anastomosis. If there is a significant size mismatch or if the vein has backflow, an end-to-side anastomosis should be performed. Successful bypass is confirmed through direct visualization or infrared imaging of the corresponding dye across the anastomotic site (Fig. 2).

Since its introduction, there have been multiple studies published with the goal of evaluating outcomes after LVB. In a prospective study of 100 cases, subjective symptomatic improvement was reported by 96% of patients, and a 42% volume reduction was seen at 12 months.^30^

LVB is most effective in the early stages of lymphedema. Long-term anastomotic patency is more achievable when lymphatic channels with minimal sclerosis and veins with low backpressure are used. Moreover, studies have shown that outcomes improve with a higher number of bypasses, suggesting that maximizing the number of bypasses performed may be advantageous.^31^

#### Vascularized Lymph Node Transplant

Vascularized lymph node transplant (VLNT) is a surgical procedure aimed at restoring lymphatic function by transplanting a healthy, functioning lymph node basin along with its blood supply from one part of the body to an area with compromised lymphatic drainage. Although the exact mechanisms are not fully understood, VLNT appears to enhance lymphatic drainage through two primary pathways.

The first is lymphangiogenesis, in which the transplanted lymph nodes secrete growth factors, particularly vascular endothelial growth factor C (VEGF-C). This stimulates the formation of new lymphatic pathways that connect with nearby lymphatic structures, enabling improved lymph outflow and drainage from the affected limb. The second, neo-lymphangiogenesis, involves the development of new lymphatico-venous drainage pathways within the transplanted lymph nodes. Driven by perfusion gradients between arterial inflow and venous outflow, this process creates a “pumping” effect that promotes fluid clearance from the limb through these new channels.^32–36^

There are several options for donor site selection in vascularized lymph node transfer, and the optimal choice will depend on the individual case. Nodal harvest from the supraclavicular, omentum, mesenteric, groin, and lateral thoracic regions have been described. When using the groin as a donor site, focusing on the lateral nodes supplied by the superficial iliac artery is recommended. This approach minimizes the risk of lower-limb lymphedema by avoiding the central and medial nodes responsible for lower limb drainage. Reverse mapping techniques can be especially helpful in these cases to avoid donor site lymphedema. In general, the senior author favors the use of the supraclavicular flap for treating lower extremity lymphedema and the lateral thoracic nodes for addressing upper extremity lymphedema.

The decision between heterotopic (placing the nodes in a different anatomical area) versus orthotopic (placing the nodes in the original lymphatic region) placement of lymph nodes depends on the location and severity of swelling in the affected limb, as well as the need for scar release. A patient with swelling primarily in the distal limb may benefit from distal placement of the transferred lymph nodes, while a patient with more widespread swelling involving the entire or proximal limb might experience better outcomes with proximal placement of the lymph nodes.^37^ Additionally, patients with secondary lymphedema who have extensive scarring and damage in the region of their nodal basins may benefit from orthotopic placement and scar release procedures, which can enhance venous drainage and significantly reduce overall swelling.

Upper extremity lymphedema often results from surgery or radiation in the axillary area. In these cases, a thorough scar release is essential to create a healthy tissue bed that supports lymphangiogenesis and to provide venous decompression. The thoracodorsal system in the axilla, the anterior recurrent ulnar artery or the radial artery at the elbow, and the radial artery at the wrist, have been described as recipient vessels in the upper extremity. A pedicled muscle-sparing latissimus dorsi flap carrying lateral thoracic nodes has also been described and is the senior author’s flap of choice when addressing upper extremity lymphedema. This flap does not require microsurgery and can be harvested with relative ease.^38^

For the lower extremity, the ankle, calf, and groin are the most common recipient sites. Like the axilla, a groin that has been previously radiated or operated on may benefit from thorough scar release. Recipient vessels in the groin include branches of the superficial femoral system below the inguinal ligament or the superficial circumflex iliac vessels above it. In the calf, the medial sural artery pedicle is easily accessible and suitable for anastomosis. When transferring lymph nodes to the ankle, options for recipient vessels include the anterior tibial, posterior tibial, and dorsalis pedis arteries. At the ankle, it’s crucial to avoid excessive tension when insetting the flap, which can be achieved through skin grafting or by incorporating a skin paddle during flap harvest.

**Fig. 3:**
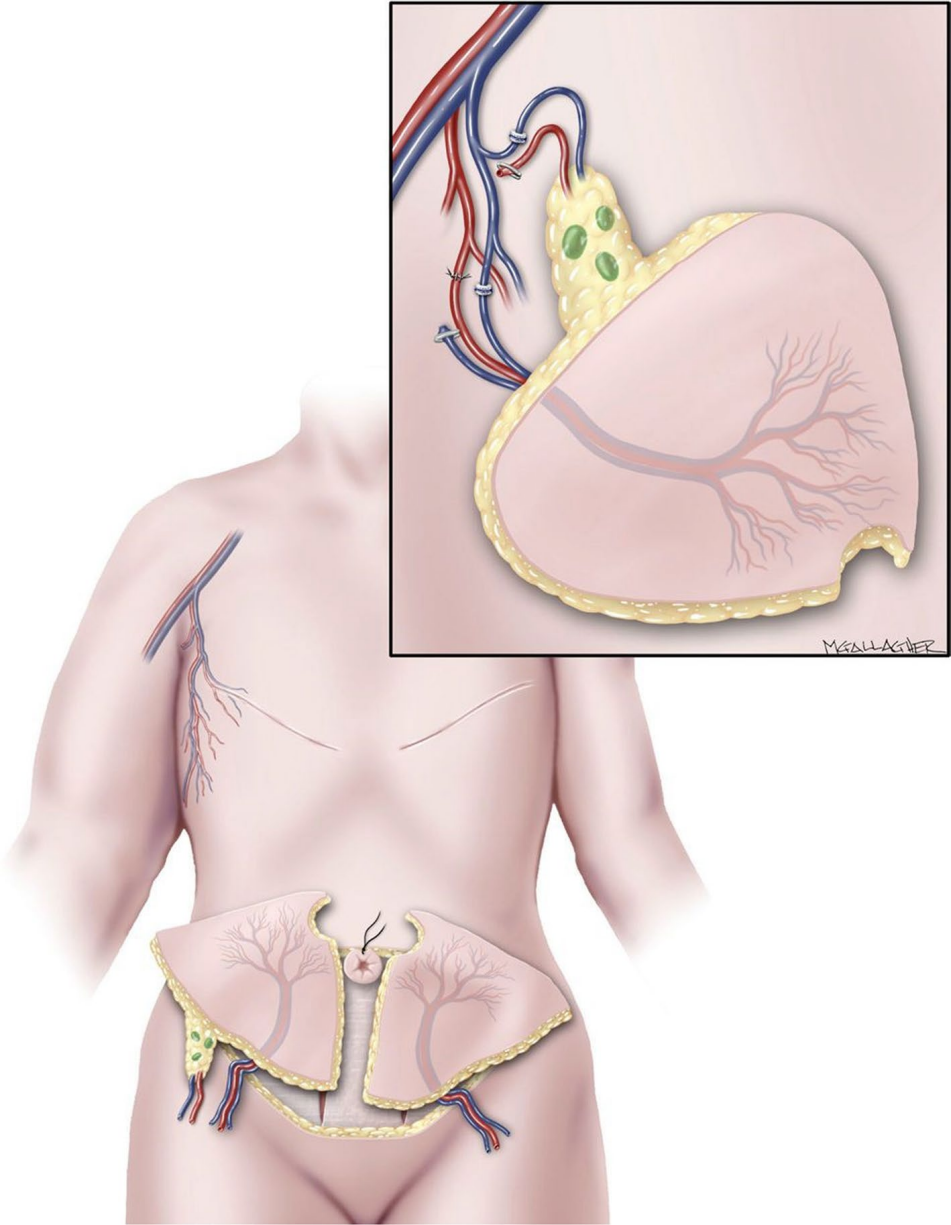
Women who are seeking breast reconstruction in combination with lymphedema treatment may benefit from combined abdominally based free flap with inguinal lymph nodes; this technique involves the harvest of abdominal tissue on the basis of the deep inferior epigastric system, along with inguinal lymph nodes harvested between the superficial inferior epigastric and superficial circumflex iliac vessels; upon transfer to the chest, the deep inferior epigastric pedicle is connected to the internal mammary system as in traditional autologous breast reconstruction cases; in addition, the superficial system is connected to the thoracodorsal vessels, which facilitates lymph node positioning in the axillary space; *source*: Nguyen AT, Chang EI, Suami H, Chang DW. An algorithmic approach to simultaneous vascularized lymph node transfer with microvascular breast reconstruction. *Ann Surg Oncol*. 2015;22(9):2919–2924. https://doi.org/10.1245/S10434-015-4408-4

Women who are seeking breast reconstruction in combination with lymphedema treatment may benefit from combined abdominally based free flap with inguinal lymph nodes. This technique involves the harvest of abdominal tissue on the basis of the deep inferior epigastric system, along with inguinal lymph nodes harvested between the superficial inferior epigastric and superficial circumflex iliac vessels (Fig. 3). Upon transfer to the chest, the deep inferior epigastric pedicle is connected to the internal mammary system as in traditional autologous breast reconstruction cases. In addition, the superficial system is connected to the thoracodorsal vessels, which facilitates lymph node positioning in the axillary space.^39^

#### Simultaneous VLNT and LVB

Recently, there has been growing evidence supporting the combined use of VLNT and LVB in the management of lymphedema. VLNT and LVB address the condition through different mechanisms and offer patients relief via distinct pathways. While LVB may provide near-immediate relief by aiding in limb decompression, VLNT requires more time to establish new lymphatic pathways at the recipient site and offer long-term, sustained benefits that take longer for the patient to fully appreciate. Results in the literature have demonstrated promising results with this technique, with improvement in limb volume and quality of life scores.^40,41^

At our institution, we prefer to perform these two procedures together whenever possible. For this reason, we recommend ICG-lymphangiography for all patients scheduled for VLNT to assess their suitability for simultaneous LVB. If the patient qualifies for bypass, this offers an additional treatment option that can help alleviate their symptoms.

### Ablative Procedures

#### Liposuction and Direct Excision

In long standing, chronic lymphedema, fluid accumulation is eventually replaced by soft tissue hypertrophy in the form of fibrosis and fat deposition. Physiological procedures become ineffective at this stage, and treatment strategies become focused on debulking of the limb through liposuction or direct excision. Suction-assisted lipectomy has been shown to reduce rates of cellulitis and improve patient quality of life. Postoperative continuous compression therapy is paramount and mandatory to maintain the effects of liposuction.^42^ It has become generally accepted that lymphedema presents on a spectrum of fluid and fibroadipose disease. In select patients with a mixed presentation of swelling, liposuction may be combined with physiological procedures to target stubborn areas of deposition and reduce dependence on continuous garment use.^43,44^ Cases of large-volume liposuction that result in skin redundancy may benefit from concurrent localized skin excision to further aid in limb reduction. However, in select patients, more extensive excisional debulking in the form of the Charles procedure may be necessary. Although rarely performed due to its significant risks and morbidity, the Charles procedure remains an option for patients with end-stage lymphedema.^45^

### Prophylactic Procedures

Lymphedema in the USA most commonly arises as a complication of oncologic therapy following lymph node dissection and/or radiation therapy. In the breast cancer population specifically, rates of lymphedema have been shown to be range of 25–35% after axillary lymph node dissection. Given the substantial morbidity associated with this condition, growing interest in strategies to reduce the risk of lymphedema at the time of lymph node dissection has become increasingly recognized by both surgeons and patients. One such approach is the lymphatic microsurgical preventative healing approach (LYMPHA), coined by Boccardo in 2003, also known as immediate lymphatic reconstruction (ILR).^46^ This technique aims to lower the likelihood of lymphedema by prophylactically bypassing one or more transected lymphatic vessels and anastomosing them to adjacent veins during the lymph node dissection procedure.

ILR has had promising results in reducing the risk of breast-cancer-related lymphedema (BCRL). Preliminary results from a randomized controlled trial conducted by Coriddi et al. revealed that ILR was associated with a 20% reduction in risk of lymphedema rates and improved quality of life scores.^47^ Additionally, a systematic review and meta-analysis conducted in 2022 further corroborated these findings with significant risk reduction of lymphedema development in patients who underwent immediate lymphatic reconstruction.^48^ Furthermore, the oncologic safety of this surgery has been reliably demonstrated with no difference in axillary or distant recurrence rates.^49,50^

Although evidence supporting ILR has been largely favorable, some patients still develop BCRL despite undergoing the procedure, and identifying those at higher risk remains uncertain. There has been literature to suggest that patients who experience lymphedema after ILR are more likely to have a higher BMI, with a BMI of 30 or greater increasing the risk by 2.6 times.^51^ This raises important questions about the optimal candidate for ILR, highlighting the need for further research to refine patient selection and improve outcomes. Key areas of investigation include whether preoperative anatomic variations serve as risk factors and whether the division of specific lymphatics contributes more significantly to lymphedema development. A deeper understanding of these factors will not only help predict which patients are most at risk, but also guide surgeons in prioritizing critical lymphatic structures during immediate reconstruction—an area of active and ongoing research.

Additionally, while ILR has been most extensively studied in patients with breast cancer, there is growing interest in its potential application for other solid tumors, such as melanoma and sarcoma, with similarly promising outcomes. However, further research is needed to validate these findings and assess the oncologic safety of the procedure in these populations.^52,53^

## Conclusions

The management of lymphedema continues to evolve, with both medical and surgical treatments playing crucial roles in improving patient outcomes. Conservative approaches, including compression therapy, manual lymphatic drainage, and exercise, remain foundational in symptom management and improving quality of life. Surgical treatments have emerged as promising options for long-term relief and functional improvement. Ongoing research and advancements in diagnostic tools are likely to further refine treatment strategies in the future.
